# From the Editor’s Desk

**Published:** 2015

**Authors:** PJ Commerford

**Affiliations:** Editor in Chief

In this issue of the Journal, Seedat (page 193) asks why the control of hypertension in sub-Saharan Africa is as bad as it is. Citing the importance of the disease as a cause of death and disability, he concludes that both the prevalence of hypertension and the failure to control it properly is driven by the poverty of the population of the region, the cost of pharmaceuticals, and a lack of medical resources. He finishes with a rousing call, echoing the late iconic President Mandela, to all of us to find African solutions to African problems.

In an editorial comment, Campbell and Legoum (page 152) re-iterate the size of the problem of hypertension in the region, and remind us that this was not always the case. Mechanisms we do not always fully understand, but which are almost certainly driven by urbanisation and poverty, are probably driving the epidemic. Furthermore, they point out that recognition and acknowledgement of the size and importance of the problem is an important first step to finding a resolution. Crucially, they discuss that at recent meetings in Africa, local leaders and champions have emerged who can spearhead an offensive on this scourge. They also list a number of organisations committed to the project. All involved in treatment and control of hypertension in Africa need to read the contributions of Seedat and Campbell.

In one of those happy moments of editorial serendipity, we are able to publish the literature review of Pinchevsky and co-authors in this same issue (page 188). These authors investigated the published results of the success of guideline directed control of a number of important risk factors (including blood pressure) in patients with diabetes. Only 35.2% (range 7.4–66.3%) of patients achieved a target blood pressure of 130/80 mmHg (or less), and targets for glycaemic and lipid control were not much better. It is interesting to note that even in well-resourced countries, achievement of targets in this vulnerable population were most unsatisfactory. Perhaps we need to re-think traditional methods of management and reflect on why we are so unsuccessful in our usual management strategies. It may be time for the world to look more closely at a polypill or alternative novel approach.

Permanent pacemaker implantation (PPMI) is a very effective tool to treat bradyarrhythmias, particularly complete heart block. The sad fact is that many patients who should receive PPMI in many parts of Africa (and I assume other similarly poorly resourced countries) do not receive the life-saving benefit and dramatic symptomatic improvement that PPMI offers. Pacemaker implantation is simple, at least for the basic ventricular-paced, ventricular-inhibited (VVI) systems that most patients with complete heart block require. The technique can be learned in a few months, it requires basic surgical skills, which most doctors possess, and access to fluoroscopy.

The challenge with pacemakers is the cost of the necessary hardware. A pacemaker generator, in its most basic form, costs US$2 500–3 000 and the leads cost US$800–1 000. The high cost of pacemakers results in limited access for deserving patients in under-resourced countries to these dramatic improvements in both quality of life and life expectancy. As outlined by Jama and colleagues (page 181), re-use of such devices is both feasible and clinically safe, provided the necessary skills for re-testing and sterilisation of the devices are available. Such programmes have been available and in place in parts of Africa for years but have not been subjected to the sort of post-implantation examination performed by Jama and co-authors, and they are to be congratulated for that.

I have purposefully not addressed implantable cardioverter defibrillator (ICD) re-use, which they also discuss. I believe that we in Africa need to be able to address the relatively simple issue of pacing for complete heart block, ensure that it is relatively easily available to all who need it, and work towards simple pacemaker availability before worrying about more complex devices, which while undoubtedly effective, offer marginal benefit compared to the wonderful benefit of VVI pacing for symptomatic complete heart block.

